# Visual Features and Their Own Optical Flow

**DOI:** 10.3389/frai.2021.768516

**Published:** 2021-12-01

**Authors:** Alessandro Betti, Giuseppe Boccignone, Lapo Faggi, Marco Gori, Stefano Melacci

**Affiliations:** ^1^ Department of Information Engineering and Mathematics, Università degli Studi di Siena, Siena, Italy; ^2^ PHuSe Lab, Department of Computer Science, Università degli Studi di Milano, Milan, Italy; ^3^ Department of Information Engineering, Università degli Studi di Firenze, Firenze, Italy; ^4^ Universitè Côte D’Azur, Inria, CNRS, I3S, Maasai, Sophia-Antipolis, France

**Keywords:** affordance, convolutional neural networks, feature flow, motion invariance, optical flow, transport equation

## Abstract

Symmetries, invariances and conservation equations have always been an invaluable guide in Science to model natural phenomena through simple yet effective relations. For instance, in computer vision, translation equivariance is typically a built-in property of neural architectures that are used to solve visual tasks; networks with computational layers implementing such a property are known as Convolutional Neural Networks (CNNs). This kind of mathematical symmetry, as well as many others that have been recently studied, are typically generated by some underlying group of transformations (translations in the case of CNNs, rotations, etc.) and are particularly suitable to process highly structured data such as molecules or chemical compounds which are known to possess those specific symmetries. When dealing with video streams, common built-in equivariances are able to handle only a small fraction of the broad spectrum of transformations encoded in the visual stimulus and, therefore, the corresponding neural architectures have to resort to a huge amount of supervision in order to achieve good generalization capabilities. In the paper we formulate a theory on the development of visual features that is based on the idea that movement itself provides trajectories on which to impose consistency. We introduce the principle of Material Point Invariance which states that each visual feature is invariant with respect to the associated optical flow, so that features and corresponding velocities are an indissoluble pair. Then, we discuss the interaction of features and velocities and show that certain motion invariance traits could be regarded as a generalization of the classical concept of affordance. These analyses of feature-velocity interactions and their invariance properties leads to a *visual field theory* which expresses the dynamical constraints of motion coherence and might lead to discover the joint evolution of the visual features along with the associated optical flows.

## 1 Introduction

Deep learning has revolutionized computer vision and visual perception. Amongst others, the great representational power of convolutional neural networks and the elegance and efficiency of Backpropagation have played a crucial role (Krizhevsky et al., 2012). By and large, there is a strong scientific recognition of their capabilities, which is very well deserved. However, an important but often overlooked aspect is that natural images are swamped by nuisance factors such as lighting, viewpoint, part deformation and background. This makes the overall recognition problem much more difficult (Lee and Soatto, 2011; Anselmi et al., 2016). Typical CNNs architectures, not structurally modelling these possible variations, require a large amount of data with high variability to gain satisfying generalization skills. Some recent works have addressed this aspect focusing on the construction of invariant (Gens and Domingos, 2014; Anselmi et al., 2016) or equivariant (Cohen and Welling, 2016) features with respect to a priori specified symmetry groups of transformations. We argue that, when relying on massively supervised learning, we have been working on a problem that is—from a computational point of view—remarkably different and likely more difficult with respect to the one offered by Nature, where motion is in fact in charge for generating visual information. Motion is what offers us an object in all its poses. Classic translation, scale, and rotation invariances can clearly be obtained by appropriate movements of a given object (Betti et al., 2020). However, the experimentation of visual interaction due to motion goes well beyond the need for these invariances and it includes the object deformation, as well as its obstruction. Could not be the case that motion is in fact nearly all we need for learning to see? Current deep learning approaches based on supervised images mostly neglect the crucial role of temporal coherence, ending up into problems where the extraction of visual concepts can only be based on spatial regularities. Temporal coherence plays a fundamental role in extracting meaningful visual features (Mobahi et al., 2009; Zou et al., 2011; Wang and Gupta, 2015; Pan et al., 2016; Redondo-Cabrera and Lopez-Sastre, 2019) and, more specifically, when dealing with video-based tasks, such as video compression (Bhaskaran and Konstantinides, 1997). Some of these video-oriented works are specifically focused on disentangling content features (constant within the selected video clip) from pose and motion features (that codify information varying over time) (Denton and Birodkar, 2017; Villegas et al., 2017; Hsieh et al., 2018; Tulyakov et al., 2018; Wang et al., 2020). The problem of learning high-level features consistent with the way objects move was also faced in Pathak et al. (2017) in the context of unsupervised object foreground versus background segmentation.

In this vein, we claim that feature learning arises mostly from motion invariance principles that turn out to be fundamental for detecting the object identity as well as characterizing interactions between features themselves. To understand that, let us start considering a moving object in a given visual scene. The object can be thought of as made up of different material points, each one with its own identity that does not change during the object motion. Consequently, the identity of the corresponding pixels has also to remain constant along their apparent motion on the retina. We will express this idea in terms of feature fields (i.e. functions of the given pixel and the specific time instant) that are invariant along the trajectories defined by their *conjugate* velocity fields, extending, in turn, the classical brightness invariance principle for the optical flow estimation (Horn and Schunck, 1981). Visual features and the corresponding optical flow fields make up an indissoluble pair linked by the motion invariance condition that drives the entire learning process. Each change in the visual features affects the associated velocity fields and vice versa. From a biological standpoint, recent studies have suggested that the ventral and dorsal pathways may not be as independent as originally thought (Milner, 2017). Following this insight, we endorse the joint discovery of visual features and the related optical flows, pairing their learning through a motion invariance constraint. Motion information does not only confer object identity, but also its affordance. As defined by Gibson in his seminal work (Gibson, 1966, 1979), affordances essentially characterize the relation between an agent and its environment and, given a certain object, correspond to the possible actions that can be executed upon it. A chair, for example, offers the affordance of seating a human being, but it can have other potential uses. In other words, the way an agent interacts with a particular object is what defines its affordance, and this is strictly related to their relative motion. Extending and generalizing this classic notion of affordance to visual features, we will define the notion of affordance field, describing the interaction between pairs of visual features. Essentially, these interactions are defined by the relative motion of the features themselves so that the corresponding affordance fields will be required to be invariant with respect to such relative motion. Hence, in the rest of the paper, we will use the term affordance in this broader sense.

This paper is organized as follows. Section 2 is focused on classical methods for optical flow estimation. In this case, the brightness is given by the input video and the goal is to determine the corresponding optical flow through the *brightness invariance condition*. Typical regularization issues, necessary to specify a unique velocity field, are also addressed. Section 3 is devoted to extend the previous approach to visual features. This time, features are not given in advance but are jointly learnt together with the corresponding velocity fields. Features and velocities are tied by the motion invariance principle. After that, the classical notion of affordance by Gibson (1966), Gibson (1979) is introduced and extended to the case of visual features. Even in this case motion invariance (with respect to relative velocities) plays a pivotal role in defining the corresponding affordance fields. At the end of Section 3 regularization issues are also considered and a formulation of learning of the visual fields is sketched out, together with the description of a possible practical implementation of the proposed ideas through deep neural networks. Finally, Section 4 draws some conclusions.

## 2 Optical Flow

The fundamental problem of optical flow estimation has been receiving a lot of attention in computer vision. In spite of the growing evidence of performance improvement (Fischer et al., 2015; Ilg et al., 2017; Zhai et al., 2021), an in-depth analysis on the precise definition of the velocity to be attributed to each pixel is still questionable (Verri, 1987; Verri and Poggio, 1989; Aubert and Kornprobst, 2006). While a simple visual inspection of some recent top level optical flow estimation systems clearly indicates remarkable performance, the definition of “optical flow” is difficult and quite slippery. Basically, we need to associate each pixel with its velocity. When considering the temporal quantization, any sound definition of such a velocity does require to know where any pixel moves on the next frame. How can we trace any single pixel? Clearly, any such pixel corresponds to a “point” of an “object” in the visual environment and the fundamental requirement to fulfill is that of tracking the point of the object.

An enlightening answer to this question was given by Horn and Shunck in a seminal paper published at the beginning of the eighties (Horn and Schunck, 1981). The basic assumption is the local-in-time constancy of *the brightness* intensity function *I*: Ω × [0, *T*] → [0, 1] where Ω is a subset of $R^{2}$ . In other words, ∀*t*_0_ > 0 there exists a *τ* > 0 such that for every *x*_0_ ∈ Ω we can define the trajectory $\gamma_{x_{0}}:\left[t_{0},t_{0}+\tau \right]\to \Omega$ that maps $t\mapsto \gamma_{x_{0}}\left(t\right)\in \Omega$ for which

$$\begin{array}{cc} & I\left(\gamma_{x_{0}}\left(t\right),t\right)=I\left(x_{0},t_{0}\right),\;\forall t\in \left[t_{0},t_{0}+\tau \right];\\ & \left(\gamma_{x_{0}}\left(t_{0}\right),t_{0}\right)=\left(x_{0},t_{0}\right). \end{array}$$ (1)

Assuming smoothness we can approximate this condition to the first order taking into account only infinitesimal temporal distances and obtain at *t* = *t*_0_:

$$\partial_{t}I\left(x_{0},t_{0}\right)+u\left(x_{0},t_{0}\right)\cdot \nabla I\left(x_{0},t_{0}\right)=0,$$ (2)

where $u\left(x_{0},t_{0}\right)≔\left(d\gamma_{x_{0}}/dt\right)\left(t_{0}\right)$ is the optical flow and ⋅ is the standard scalar product in $R^{2}$ .

**FIGURE 1:**
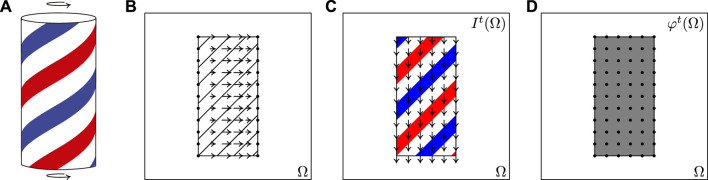
Barber’s pole example. **(A)** The 3-D object spinning counterclockwise. **(B)** The 2-D projection of the pole and the projected velocity on the retina Ω. **(C)** The brightness of the image and its optical flow pointing downwards. **(D)** A feature map that responds to the object and its conjugate (zero) optical flow.

Assumption Eq. 1 is reasonable when there are no occlusions and changes of the light source are assumed to be “small.” Of course, in real world applications of computer vision these scenarios are not always met. On the other hand, it is clear that the optical flow *u* could be derived from an invariance condition of the type Eq. 1 applied to different and possibly more “stable” visual features rather than to the brightness itself. As shown in Figure 1, this would give a different optical flow with respect to the one defined through the brightness invariance condition (Figure 1C). For example, a feature responding to the entire barber’s pole, that is standing still, would have an associated optical flow that is null everywhere (Figure 1D). Still, we have to keep in mind that in both cases the resulting optical flow is different from the 2-D motion field (defined as the projection on the image plane of the 3-D velocity of the visual scene, see e.g. Aubert and Kornprobst (2006)) shown in Figure 1B.

This indeed is the main motivation to couple the problem of feature extraction together with motion invariance constraints and the derivation of robust and meaningful optical flows associated to those visual features.

### 2.1 Regularization of the Optical Flow

Before going on to lay out the theory for the extraction of motion invariant visual features, we need to recall some facts about the optical flow condition Eq. 2. Given a video stream described by its brightness intensity *I* as it is defined as in Section 1, the problem of finding for each pixel of the frame spatial support at each time instant the velocity field *u*(*x*, *t*) satisfying

$$\partial_{t}I\left(x,t\right)+u\left(x,t\right)\cdot \nabla I\left(x,t\right)=0\;\forall \left(x,t\right)\in \Omega \times \left[0,T\right]$$ (3)

is clearly ill posed since a scalar equation is not sufficient to properly constrain the two components of *u*. Locally, we can unequivocally determine only the component of *u* along ∇*I*.

Although many methods have been proposed to overcome this issue (see for example the work of Aubert and Kornprobst (2006)), that is usually referred to as the *aperture problem*, here we are interested in the class of approaches that aims at regularizing the optical flow:

$$\underset{v}{\inf}\left(A_{I}\left(v\right)+S\left(v\right)+H_{I}\left(v\right)\right)$$ (4)

where *A*_*I*_ is a functional that enforces constraint Eq. 3, hence a standard choice is

$$A_{I}\left(v\right)≔\int_{\Omega }{\left(\partial_{t}I\left(x,t\right)+v\cdot \nabla I\left(x,t\right)\right)}^{2}\;dx,$$ (5)

*S* imposes smoothness and *H* may be used to condition the extraction of the flow over spatially homogeneous regions. Depending on the regularity assumptions on *I* and the properties that we want to impose on the solution of this regularized problem (namely if we want to admit solutions that preserve discontinuities or not) the exact form of *S* and the form and presence of *H*_*I*_ may vary. For example, in the original approach proposed in Horn and Schunck (1981) we find:

$$\text{Horn–Schunck regularization}:S\left(v\right)=\int_{\Omega }|\nabla v^{1}{|}^{2}+|\nabla v^{2}{|}^{2}dx,\;H_{I}\equiv 0.$$ (6)

Note that since we are interested in extracting the optical flow for any frame of a video, namely the field *u*(*x*, *t*), it is useful for any function $f:\Omega \times \left[0,T\right]\to R^{p}$ to define $f^{t}:\Omega \to R^{p}$ as *f* ^*t*^(*x*)≔*f*(*x*, *t*). With this notation, when the infimum in Eq. 4 is attained, we can pose.

$$u\left(x,t\right)=u^{t}\left(x\right)=\underset{v}{\arg\;\min}\left(A_{I}\left(v\right)+S\left(v\right)+H_{I}\left(v\right)\right).$$ (7)

**FIGURE 2:**
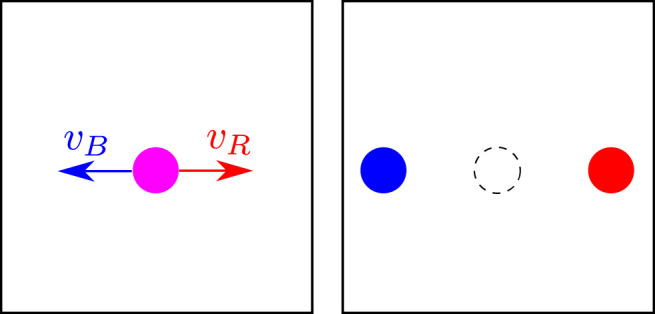
Tracking of different color components in a synthetic example. In this case, each color component is associated to a specific velocity field.

Notice that the brightness might not necessarily be the ideal signal to track. Since the brightness can be expressed as a weighed average of the red *R*, green *G*, and blue *B* components, one could think of tracking each single color component of the video signal by using the same invariance principle stated by Eq. 3. It could in fact be the case that one or more of the components *R*, *G*, *B* are more invariant in the sense of Eq. 3 during the motion of the corresponding material point, see Figure 2. In that case, in general, each color can be associated with corresponding optical flows *v*_*R*_, *v*_*G*_, *v*_*B*_ that might differ. In doing so, instead of tracking the brightness, one can track the single colors. Instead, under the assumption that each color component has the same optical flow *v* = *v*_*R*_ = *v*_*G*_ = *v*_*B*_ we have.

$$\frac{\partial }{\partial t}\left(\begin{array}{c} R\\ G\\ B \end{array}\right)+v\cdot \nabla \left(\begin{array}{c} R\\ G\\ B \end{array}\right)=0,$$ (8)

where *v* ⋅∇(*R*,*G*,*B*)′≔(*v* ⋅∇*R*,*v* ⋅∇*G*,*v* ⋅∇*B*)′. It is worth mentioning that the simultaneous tracking of different channels might contribute to a better positioning of the problem since, in general, rank∇(*R*, *G*, *B*)′ = 2 and the system Eq. 8 admits an unique solution.^1^ One can think of the color components as features that, unlike classical convolutional spatial features, are temporal features.

**FIGURE 3:**
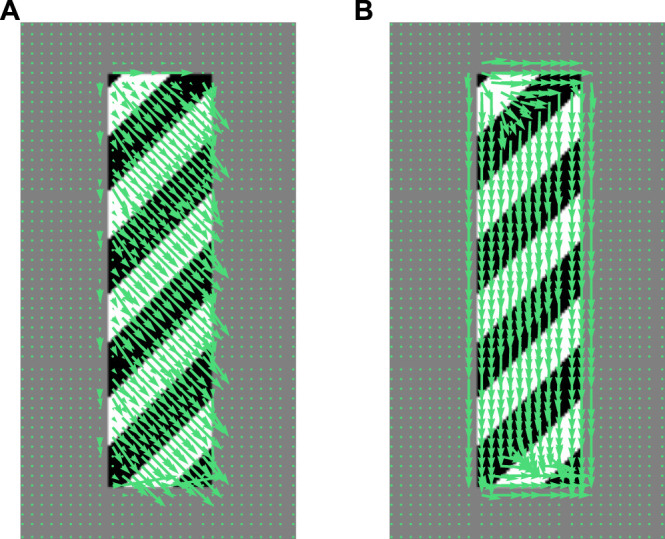
Barber’s pole optical flow (sub-sampled). **(A)** Horn–Schunck method (Horn and Schunck, 1981) with smoothing factor coefficient = 1 **(B)** Gunnar Farnebäck’s algorithm (Farnebäck, 2003) in the quadratic expansion case.

Before proceeding further, let us underline that some other optical flow methods try to directly solve the brightness invariance condition Eq. 1 without differentiating it. This is the case, for example, of the Gunnar Farnebäck’s algorithm (Farnebäck, 2003): the basic idea here is to approximate the brightness of the input images through polynomial expansions with variable coefficients, and the brightness invariance condition Eq. 1 is then solved under this assumption. Figure 3 shows the optical flows extracted by the Horn-Schunck and Farnebäcks methods in the barber’s pole case.

In the next section, we will discuss how to use a very similar approach, based on the consistency of features along apparent motion trajectories on the frame spatial support, to derive visual features *φ*_*i*_ along with the corresponding *conjugate* optical flows $v_{\varphi_{i}}$ . We anticipate that this motion consistency condition will also play a prominent role in defining affordance features, as described in Section 3.2.

## 3 Feature Extraction and Conjugate Velocities

As we have already anticipated in the previous sections, the optical flow extracted by imposing an invariance condition like the one in Eq. 3 strongly depends on the features on which we are imposing that invariance; hence it should be not surprising that different sets of features could give rise to different optical flows. This can be easily understood by considering the barber’s pole example in Figure 1. The related classical optical flow is depicted in Figure 1C, see also Figure 3, and it is different from the projection of the 3-D velocities on the frame spatial support Figure 1B (the resulting optical flow is an optical illusion indeed). Let us now assume the existence of a visual feature *φ*_*r*_ characterizing the red stripes, that is *φ*_*r*_(*x*, *t*) = 1 iff (*x*, *t*) is inside a stripe. As the barber’s pole rotates, the conjugate velocity $v_{\varphi_{r}}$ is, in this simplified case, the same that one would have obtained from the brightness invariance condition Eq. 3. An additional level of abstraction can be gained when looking at the whole object. Again, we are assuming the existence of an higher level visual feature *φ*_*object*_ characterizing it. Then, considering that the barber’s pole is standing still, the velocity field associated to that feature is everywhere null, as shown in Figure 1D.

This example clearly explains how different velocity fields can be associated to different visual features, but we still have to go one step further. Until now, mimicking the case of the classical optical flow estimation given the corresponding input brightness, we have described the construction of velocity fields starting from visual features whose existence was a priori assumed. Recent studies have suggested that the ventral and dorsal pathways may not be as independent as originally thought. Evidence for contributions from ventral stream systems to the dorsal stream indicates a crucial role in mediating complex and flexible visuomotor skills. Meanwhile, complementary evidence points to a role for posterior dorsal-stream visual analysis in certain aspects of 3-D perceptual function in the ventral stream (but see Milner, 2017 for a review). As pointed out by Milner (2017) potential cross-stream interactions might take three forms:

1) Independent processing: computations along the separate pathways proceed independently and in parallel and reintegrate at some final stage of processing within a shared target brain region; this might be achieved via common projections to the lateral prefrontal cortex or superior temporal sulcus (STS);
2) Feedback: processing along the two pathways is modulated by the existence of feedback loops which transmit information from downstream brain regions, including information processed along the complementary stream; feedback is likely to involve projections to early retinotopic cortical areas.
3) Continuous cross-talk: information is transferred at multiple stages and locations along the two pathways.

The three forms need not be mutually exclusive and a resolution of the problems of visual integration might involve a combination of such possibilities (Milner, 2017).

Yet, from a learning standpoint, the cross-talk mode is intriguing for setting some minimal conditions for an agent (either biological or artificial) in order to develop visual capabilities. Following this biological insight, we endorse the indissoluble conjunction of features and velocities and, consequently, their joint discovery based on the motion invariance condition.

$$\partial_{t}\varphi_{i}\left(x,t\right)+v_{\varphi_{i}}\left(x,t\right)\cdot \nabla \varphi_{i}\left(x,t\right)=0\forall \left(x,t\right)\in \Omega \times \left[0,T\right],\forall \;i=1,\ldots ,d,$$ (9)

where we are considering *d* different visual features. Locally, this equation means that, at each pixel *x* of the frame spatial support and specific time instant *t*, features *φ*_*i*_ are preserved along the trajectories defined by the corresponding velocity fields $v_{\varphi_{i}}$ and starting at (*x*, *t*). An object clearly does not change its identity while it is moving. Consequently, the identity of the corresponding pixels on the frame spatial support has to remain invariant along the apparent motion defined by the associated optical flows. Thinking of the brightness as the simplest visual feature based on single pixels, Eq. 9 correctly reduces to the brightness invariance condition Eq. 3. Notice that if there is no optical flow for a given pixel $\bar{x}$ , that is, if $v_{\varphi }\left(\bar{x},t\right)=0$ for all *t* ∈ [0, *T*], then $\varphi_{t}\left(\bar{x},t\right)=0$ . This means that the absence of the optical flow in $\bar{x}$ results into $\varphi \left(\bar{x},t\right)=c_{\varphi }$ for all *t* ∈ [0, *T*], which is the obvious consistency condition that one expects in this case. Likewise, a constant field *φ*(*x*, *t*) in a subregion $C\subset \Omega \times \left[0,\;T\right]$ makes Eq. 9 satisfied on *C* independently of *v*_*φ*_.

Like for the brightness, in general, the invariance condition Eq. 9 generates an ill-posed problem. In particular, when the moving object has a uniform color, we can notice that brightness invariance holds for virtually infinite trajectories. Likewise, any of the features *φ* is expected to be spatially smooth and nearly constant in small portions of the frame spatial support, and this restores the ill-posedness of the classical problem of determining the optical flow that has been addressed in the previous section. Unlike brightness invariance, in the case of visual features the ill-posedness of the problem has a double face. Just like in the classic case of estimating the optical flow, *v*_*φ*_ is not uniquely defined (the aperture problem). On top of that, now the corresponding feature *φ* is not uniquely defined, too. We will address regularization issues in Section 3.3 where, including additional information other than coherence on motion trajectories, we will make the learning process well-posed. Of course, the regularization process will also involve a term similar to the one invoked for the optical flow *v*, see Eq. 6, that will be imposed on *v*_*φ*_. Given what we have discussed so far, we can also expect the presence of some regularization term concerning the features themselves and their regularity. Finally, these terms will be complemented with an additional “prediction” index necessary to avoid trivial features’ solutions (we postpone its description to Section 3.3).

The basic notion at the core of this section is that $\left\{\left(\varphi_{i},v_{\varphi_{i}}\right)\right\}$ can be treated as indissoluble pairs bounded by the motion invariance condition that steers the entire learning process. The structure of each *φ*_*i*_ affects the associated velocity $v_{\varphi_{i}}$ and vice versa—it is therefore natural to pair their learning. Leaving aside for the moment regularization issues of Section 3.3, learning is based on a functional generalizing to Eq. 5, that is

$$A\left(\left\{\varphi_{i},\;v_{\varphi_{i}}\right\}\right)≔\frac{1}{2}\underset{i}{\sum }\int_{\Gamma }{\left(\partial_{t}\varphi_{i}\left(x,t\right)+v_{\varphi_{i}}\left(x,t\right)\cdot \nabla \varphi_{i}\left(x,t\right)\right)}^{2}d\mu \left(x,t\right),$$ (10)

where Γ = Ω × [0, *T*] and *μ* is an appropriately weighted Lebesgue measure on $R_{x}^{2}\times R_{t}$ ; its exact form defines the dynamics of the learning process itself. The minimization of such functional (plus the additional regularitation terms) is expected to return the pairs $\left(\varphi_{i},v_{\varphi_{i}}\right)$ satisfying the motion consistency condition Eq. 9. Sometimes, in what follows and when the notation is clear from the context, we will drop the subscript *φ* of *v*_*φ*_ so that $v_{\varphi_{i}}$ will be denoted as *v*_*i*_.

### 3.1 Feature Grouping

As already noticed, when we consider color images, what is done in the case of brightness invariance can be applied to the separated components R,G,B. Interestingly, for a material point of a certain color, given by a mixture of the three components, we can establish the same brightness invariance principle, since those components move with the same velocity. Said in other words, there could be group of different visual features *φ*_*i*_, *i* = 1, …, *m* that share the same velocity (*v*_*i*_ = *v*) and are consistent with it, that is *∂*_*t*_ *φ*_*i*_ + *v* ⋅∇*φ*_*i*_ = 0 ∀*i* = 1, …, *m*. Thus, we can promptly see that any feature *φ* of span(*φ*_1_, *…*, *φ*_*m*_) is still conjugated with *v*; we can think of span(*φ*_1_, *…*, *φ*_*m*_) as a functional space conjugated with *v*.

Let us now consider the feature group $\phi ={\left(\varphi_{1},\ldots ,\varphi_{m}\right)}^{\prime }$ and the corresponding invariance condition.

$$\partial_{t}\phi +v\cdot \nabla \phi =0,$$ (11)

where $\nabla \phi \in R^{m\times 2}$ is the matrix with elements ${\left(\nabla \phi \right)}_{ij}={\left(\nabla \phi_{i}\right)}_{j}$ and $v\cdot \nabla \phi ≔{\left(v\cdot \varphi_{1},\;\ldots ,\;v\cdot \varphi_{m}\right)}^{\prime }$ . An important observation, very related to the discussion about color tracking of Eq. 8, is the following one. Notice that, if we consider the case in which the only scalar feature we are dealing with is the brightness, then Eq. 11 boils down to a single equation with two unknowns (the velocity components). Differently, in the case of the feature group *ϕ*, we have *m* equations and still two unknowns. The dimension *m* of matrix ∇*ϕ* can enforce the increment of its rank, which leads to a better posedness of the problem of estimating the optical flow *v*. Because of the two-dimensional structure of the frame spatial support, which leads to $v\in R^{2}$ , and since $\nabla \phi \in R^{m\times 2}$ , with *m* ≥ 2, it turns out that feature grouping regularizes the velocity discovery. In order to understand the effect of feature grouping we can in fact simply notice that, under the assumption rank∇*ϕ* = rank(∇*ϕ*∣ − *ϕ*_*t*_), a random choice of the features yields rank∇*ϕ* = 2. As a consequence, by Rouché-Capelli theorem, linear Eq. 11 admits a unique solution in *v*. However, this regularization effect of feature grouping does not prevent ill-posedness, since *ϕ* is far from being a random map. On the opposite, it is supposed to extract a uniform value in portions of the frame spatial support that are characterized by the same feature. Hence, rank∇*ϕ* = 1 is still possible whenever the features of the group are somewhat dependent.

Feature groups, that are characterized by their common velocity, can give rise to more structured features belonging to the same group. This can promptly be understood when we go beyond linear spaces and consider for a set of indices F .

$$\left\{\begin{array}{cc} & \alpha =\underset{j\in F}{\sum }w_{j}\varphi_{j}\\ & \eta =\sigma \left(\alpha \right). \end{array}\right.$$ (12)

Evaluating *∂*_*t*_ *η* + *v* ⋅∇*η* we obtain indeed

$$\partial_{t}\eta +v\cdot \nabla \eta =\sigma^{\prime }\left(\alpha \right)\left(\partial_{t}\alpha +v\cdot \nabla \alpha \right)=\sigma^{\prime }\left(\alpha \right)\underset{j\in F}{\sum }\omega_{j}\left(\partial_{t}\varphi_{j}+v\cdot \nabla \varphi_{j}\right).$$ (13)

and we conclude that if ∀j∈F we have *∂*_*t*_ *φ*_*j*_ + *v* ⋅∇*φ*_*j*_ = 0 then also the feature *η* defined by Eq. 12 is conjugated with *v*, that is *∂*_*t*_ *η* + *v* ⋅∇*η* = 0. However, the vice versa does not hold true. Basically, the inheritance of conjugation with *v* holds in the direction towards more abstract features. Of course, the feedforward-like recursive application of the derivation stated by Eq. 12 yields a feature that is still conjugated with *v*.

### 3.2 Affordance-Related Features

Any learning process that relies on the motion of a given object can only aspire to discover the identity of that object, along with its characterizing visual features such as pose and shape. The motion invariance process is in fact centered around the object itself and, as such, it does reveal its own features in all possible expositions that are gained during motion. Humans, and likely most animals, also conquer a truly different understanding of visual scenes that goes beyond the conceptualization with single object identities. In the early Sixties, James J. Gibson coined the notion of affordance in (Gibson, 1966), even though a more refined analysis came later in (Gibson, 1979). In his own words: *“The affordances of the environment are what it offers the animal, what it provides or furnishes, either for good or ill. The verb to afford is found in the dictionary, the noun affordance is not. I have made it up. I mean by it something that refers to both the environment and the animal in a way that no existing term does. It implies the complementarity of the animal and the environment.”* Considering this animal-centric view, we gain the understanding that affordance can be interpreted as what characterizes the “interaction” between animals and their surrounding environment. In more general terms, the way an agent interacts with a particular object is what defines its affordance, and this is strictly related to their relative motion. In the last decades, computer scientists have also being working on this general idea, trying to quantitatively implement it in the fields of computer vision and robotics (Ardón et al., 2020; Hassanin et al., 2021). As far as visual affordance is concerned, that is, extracting affordance information from still images and videos, different cognitive tasks have been considered so far, as for example affordance recognition and affordance segmentation, see (Hassanin et al., 2021) for a recent review.

**FIGURE 4:**
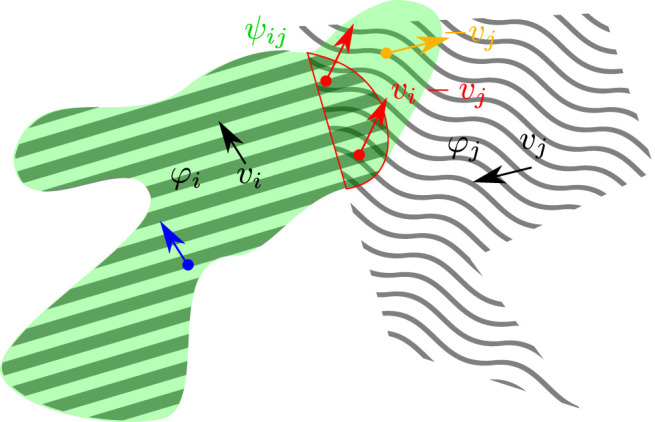
Illustration of Eq. 15 with *b*_*j*_ = − 1. The two considered features *φ*_*i*_ (diagonal lines), *φ*_*j*_ (wavy lines) translate over the frame spatial support with uniform velocities *v*_*i*_, *v*_*j*_. The green area represents where *ψ*_*ij*_ is on, while the red border identifies the region where *φ*_*i*_ and *φ*_*j*_ overlap. On the overlapping region the velocity fields of the two features are both present and here the affordance field *ψ*_*ij*_(*x*, *t*) is constrained to be consistent along the direction *v*_*i*_ − *v*_*j*_ (red arrows). Outside and on the left of the red border, the consistency term in Eq. 15 essentially collapses to the feature identity constraint Eq. 9 defined by the invariance motion property with respect to *v*_*i*_ (blue arrow). Finally, in those region where *v*_*i*_ = 0, motion consistency of *ψ*_*ij*_ is required along − *v*_*j*_ (orange arrow).

In the spirit of the previous section, we will consider a more abstract notion of affordance, characterizing the interaction between different visual features along with their corresponding conjugate velocity fields. We will focus our attention on actions that are perceivable from single pictures^2^ and on the related local notion of affordance, that will be defined by some function characterizing the interaction between feature *φ*_*j*_ and feature *φ*_*i*_ when considering the pixel *x* at the specific time instant *t*. As we will see, the principle of motion invariance can be extended to naturally define (explicitly or implicitly) this generalized notion of affordance. A natural choice is to consider what we will denote as the *affordance field ψ*_*ij*_ as a function of space and time. To implicitly codify the interaction between features *i* and *j*, *ψ*_*ij*_(*x*, *t*) has to be constrained by some relation of the form $g\left(\psi_{ij},\partial_{t}\psi_{ij},\nabla \psi_{ij},\left(\varphi_{i},v_{i}\right),\left(\varphi_{j},v_{j}\right)\right)=0$ , where we are considering only first order derivatives of the affordance field and *g* is a scalar function. In the lower order approximation (we also need quadratic terms to build scalars from vectors):

$$g\left(\psi_{ij},\partial_{t}\psi_{ij},\nabla \psi_{ij},\left(\varphi_{i},v_{i}\right),\left(\varphi_{j},v_{j}\right)\right)=a_{1}+a_{2}\psi_{ij}+a_{3}\varphi_{i}+a_{4}\varphi_{j}+a_{5}\partial_{t}\psi_{ij}+a_{6}v_{i}\nabla \psi_{ij}+a_{7}v_{j}\nabla \psi_{ij},$$ (14)

where *a*_1_, …, *a*_7_ are scalars. Considering the case in which the motion field associated to feature *φ*_*j*_ is everywhere null, the affordance field *ψ*_*ij*_ will codify a property only related to *φ*_*i*_ itself and strictly related to its identity, that has to be invariant with respect to *v*_*i*_. Thus, from this observation, we can infer *a*_1_ = *a*_2_ = *a*_3_ = *a*_4_ = 0 and *a*_5_ = *a*_6_ so that the above constraint becomes:

$$\partial_{t}\psi_{ij}+\left(v_{i}+b_{j}v_{j}\right)\cdot \nabla \psi_{ij}=0$$ (15)

where *b*_*j*_ = *a*_7_/*a*_5_. Requiring *b*_*j*_ = −1 this constraint assumes a very reasonable physical meaning, that is the motion invariance of the affordance field *ψ*_*ij*_ in the reference of feature *φ*_*j*_. Within this choice, the affordance field is conjugated with the velocity *v*_*i*_ − *v*_*j*_ indeed, which is in fact the relative velocity of feature *φ*_*i*_ in the reference of feature *φ*_*j*_. Considering points at the border of *φ*_*i*_, this can lead to slightly expand *ψ*_*ij*_ outside the region defined by *φ*_*i*_ itself, as shown in Figure 4. In the case *v*_*i*_ = 0, the motion consistency is forced “backward” along the pixels’ trajectories defined by − *v*_*j*_. In the case *b*_*j*_ = 1 Eq. 15 becomes symmetric under permutations instead so that *ψ*_*ij*_ and *ψ*_*ji*_ will be developed exploiting the same constraint. This will likely result in the same affordance feature unless some other factor (let us think for example to different initializations in neural architectures) breaks that symmetry. From the classic affordance perspective this is not a desirable property as we can easily understand considering, for example, a knife that is used to slice bread: the affordance transmitted by the knife to the bread would be strictly related to the possibility of being cut or sliced, that is clearly a property that could not be attached to the knife.

**FIGURE 5:**
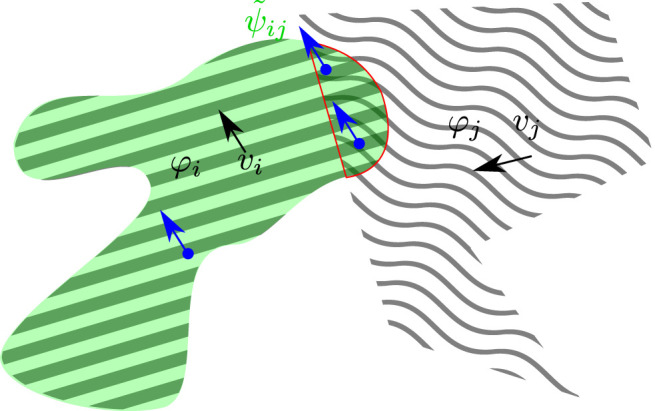
Illustration of Eq. 16. The two considered features *φ*_*i*_ (diagonal lines), *φ*_*j*_ (wavy lines) translate over the frame spatial support with uniform velocities *v*_*i*_, *v*_*j*_. The green area represents where ${\tilde{\psi }}_{ij}$ is on, while the red border identifies the region where *φ*_*i*_ and *φ*_*j*_ overlap. In this case, the motion invariance property of the affordance feature ${\tilde{\psi }}_{ij}$ is the same of the original feature field $\varphi_{i}\left(x,t\right)$ . Blue arrows identify the direction along which motion coherence of ${\tilde{\psi }}_{ij}$ is required.

Another viable and different alternative to codify the interaction between features may be the one of directly evaluating the affordance as function of the feature fields and their respective velocities: ${\tilde{\psi }}_{ij}\left(x,t,\left(\varphi_{i},v_{i}\right),\left(\varphi_{j},v_{j}\right)\right)$ . Here, we are giving up the previous field theory approach, being explicitly codifying the interaction between features in the computational scheme of the ${\tilde{\psi }}_{ij}$ function. On the other hand, since we have already distinguished ${\tilde{\psi }}_{ij}$ and *φ*_*i*_ by the different computational structure, requiring the same motion invariance property of *φ*_*i*_ with respect to *v*_*i*_ for the affordance function ${\tilde{\psi }}_{ij}$ appears a very natural choice, see also Figure 5:

$$\partial_{t}{\tilde{\psi }}_{ij}+v_{i}\cdot \nabla {\tilde{\psi }}_{ij}=0.$$ (16)

Given the possible great variability of velocity fields in a visual scene, let us underline that within this second approach some problems in the learning of the affordance function ${\tilde{\psi }}_{ij}\left(x,t,\left(\varphi_{i},v_{i}\right),\left(\varphi_{j},v_{j}\right)\right)$ may emerge. Moreover, to pursue the fascinating idea to describe all the visual processes entirely through visual fields defined on the frame spatial support, in the following we will only consider the affordance field *ψ*_*ij*_(*x*, *t*) and the related motion invariance property Eq. 15 with *b*_*j*_ = −1.

Given a certain visual environment we can easily realize that, as time goes by, object interactions begin obeying statistical regularities and the interactions of feature *φ*_*i*_ with the others become very well defined. Hence, the notion of *ψ*_*ij*_ can be evolved towards the *inherent affordance ψ*_*i*_ of feature *φ*_*i*_, which is in fact a property associated with *φ*_*i*_ while living in a certain visual environment. For example, thinking in terms of the classic notion of affordance, when considering a knife the related inherent affordance property is gained by being manipulated, in a certain way, by a virtually unbounded number of different people. Based on Eq. 15 (*b*_*j*_ = −1) we define the inherent feature affordance as the function *ψ*_*i*_(*x*, *t*) which satisfies.

$$\partial_{t}\psi_{i}+\left(v_{i}-v_{j}\right)\cdot \nabla \psi_{i}=0,\;\;1\leq i,j\leq n.$$ (17)

Let us note that the above formula can also be interpreted as the motion invariance property of *ψ*_*i*_ with respect to the velocity $v_{i}-\sum_{j=1}^{n}v_{j}/n$ . The identification feature *φ*_*i*_ pairs with the corresponding affordance feature *ψ*_*i*_, and the visual scene turns out to be effectively described by the collection of visual fields $V=\left\{\left(\varphi_{i},\psi_{i},v_{i}\right)\right\}$ . In a sense, *ψ*_*i*_ can be thought of as the abstraction of *φ*_*i*_, as it arises from its environmental interactions. A few comments are in order concerning these visual fields.

• The pairing of *φ*_*i*_ and *ψ*_*i*_ relies on the same optical flow which comes from *φ*_*i*_ *.* This makes sense, since the inherent affordance is a feature that is expected to gain abstraction coming from the interactions with other features, whereas the actual optical flow can only come from identifiable entities that are naturally defined by *φ*_*i*_ *.*
• The inherent affordance features still bring with them a significant amount of redundant information. This can be understood when considering especially high level features that closely resemble objects. For example, while we may have many different chairs in a certain environment, one would expect to have only a single concept of chair. On the opposite, *ψ* assigns many different affordance variables that are somewhat stimulated by a specific identifiable feature. This corresponds to thinking of these affordance features as entities that are generated by a corresponding identity feature.
• The collection of visual fields V is the support for high-level decisions. Of course, the recognition of specific objects does only involve the field *φ*_*i*_, whereas the abstract affordance semantic labeling is supported by features *ψ*_*i*_ *.*

In order to abstract the notion of affordance even further we can, for instance, proceed as follows: for each *κ* = 1,*…*, *n* we can consider another set of fields $\chi_{\kappa }:\Gamma \to R$ each of which satisfies the following condition

$$\partial_{t}\chi_{\kappa }+v_{j}\cdot \nabla \chi_{\kappa }=0,\;j=1,\ldots ,n.$$ (18)

In this way the variables *χ*_*κ*_ do not depend, unlike for *ψ*, on a particular *v*_*i*_, which contributes to lose the link with its firing feature. Moreover, they need to take into account, during their development, multiple motion fields which results in a motion invariant property with respect to the average velocity $\sum_{j=1}^{n}v_{j}/n$ and in a greater level of abstraction.

Once the set of the *χ*_*κ*_ is given, a method to select the most relevant affordances could simply be achieved through a linear combination. In other words, a subselection of *χ*_1_, *…*, *χ*_*n*_ can be performed by considering for each *l* = 1, *…*, *n*_*χ*_ < *n* the linear combinations

$$X_{l}≔\overset{n}{\underset{\kappa =1}{\sum }}a_{l\kappa }\chi_{\kappa },$$ (19)

where $\left(a_{l\kappa }\right)\in R^{n_{\chi }\times n}$ is a matrix of learnable parameters. Notice that since *X*_*l*_ ∈ span(*χ*_1_, *…*, *χ*_*n*_), as we remarked in Section 3.4, then *∂*_*t*_ *X*_*l*_ + *v*_*j*_ ⋅∇*X*_*l*_ = 0 for all *j* = 1, *…*, *n*. It is worth mentioning that the learning of coefficients *a*_*lκ*_ does not involve motion invariance principles. Interestingly, they can be used for additional developmental steps like that of object recognition. For example, they can be learned under the classic supervised framework along with the correspondent regularization.

### 3.3 Regularization Issues

We have already discussed the ill-posed definition of features conjugated with their corresponding optical flow. Interestingly, we have also shown that a feature group $\phi ={\left(\varphi_{i},\ldots ,\varphi_{m}\right)}^{\prime }$ with conjugate velocity *v* exhibits an inherent regularization that, however, does not prevent ill-positioning, especially when one is interested in developing abstract features that are likely constant over large regions of the frame spatial support.

Let us assume that we are given *n* feature groups *ϕ*_*i*_, *i* = 1, …*n*, each one composed of *m*_*i*_ single features (*m*_*i*_-dimensional feature vector) $\phi_{i}\in R^{m_{i}}$ . Furthermore, let *v*_*i*_ be the velocity field shared by each component of feature group *ϕ*_*i*_ and let us also denote with ***ϕ*** = (*ϕ*_1_, *…*, *ϕ*_*n*_) and with ***v*** = (*v*_1_, *…*, *v*_*n*_). We can then impose the following generalization of the smoothness term (6), used for the classical optical flow estimation, to the velocities and the corresponding visual features:

$$E=\frac{1}{2}\overset{n}{\underset{i=1}{\sum }}\int_{\Gamma }\left({\left\|\nabla v_{i}\left(x,t\right)\right\|}^{2}+\lambda_{\varphi }{\left|\phi_{i}\left(x,t\right)\right|}^{2}+\lambda_{\nabla }{\left\|\nabla \phi_{i}\left(x,t\right)\right\|}^{2}\right)\;d\mu \left(x,t\right).$$ (20)

Here, the notation ${\left\|Z\right\|}^{2}$ (generic argument matrix *Z*) means $\sum_{i,j}Z_{ij}^{2}$ , while *λ*_*φ*_, *λ*_∇_ are positive constants that express the relative weight of the regularization terms. First of all, notice that *E* is a functional of the pairs {(*ϕ*_*i*_, *v*_*i*_)}, that is, once they are given, we can compute *E*(***ϕ***, ***v***). On the contrary, the index used to regularize the classical Horn–Schunck optical flow Eq. 6 only depends on *v*. The dependence on visual features and their temporal dynamic in Eq. 20 is explained considering that, while the brightness is given, the features are learned as time goes by, which is just another facet of the feature-velocity conjugation. Moreover, it is worth mentioning that *E* only involves spatial smoothness whereas it doesn’t contain any time regularization term. There is also another difference with respect to the classic optical flow regularization Eq. 6, that is the penalizing term $\left(1/2\right){\left|\phi_{i}\right|}^{2}$ which favors the development of *ϕ*_*i*_ = 0. Of course, there is no such requirement in classic optical flow since, as already stated, the brightness is given. On the opposite, the discovery of visual features is expected to be driven by motion information, but their “default value” is expected to be null. We can promptly see that the introduction of the regularization term Eq. 20 does not suffice to achieve a well-posed learning problem. The motion invariance condition Eq. 9 is still satisfied by the trivial constant solution *φ* = *c*_*φ*_ indeed.

Important additional information comes from the need of exhibiting the human visual skill of reconstructing pictures from our symbolic representation. At a certain level of abstraction, the features that are gained by motion invariance possess a certain degree of semantics that is needed to interpret the scene. However, visual agents are also expected to deal with actions and react accordingly. As such, a uniform cognitive task that visual agents are expected to carry out is that of predicting what will happen next, which is translated into the capability of guessing the next incoming few frames in the scene. We can think of a predictive computational scheme based on the *φ*_*i*_ codes $y\left(x,t\right)=\alpha_{y}\left(\phi \left(\cdot ,t\right),t\right)\left(x\right)$ , where $\alpha_{y}:{\left(R^{\Omega }\right)}^{n}\times \left[0,T\right]\to R^{\Omega }$ and the prediction *y* needs to satisfy the condition established by the index

$$R=\frac{1}{2}\int_{\Gamma }{\left(y\left(x,t\right)-I_{t}\left(x,t\right)\right)}^{2}\;d\mu \left(x,t\right).$$ (21)

Of course, as the visual agent gains the capability of predicting what will come next, it means that the developed internal representation based on features ***ϕ*** cannot correspond with the mentioned trivial solution. Interestingly, it looks like visual perception does not come alone: the typical paired skill of visual prediction that animals exhibit helps regularizing the problem of developing invariant features. Clearly, the *φ* = *c*_*φ*_ which satisfies motion invariance is no longer acceptable since it does not reconstruct the input. This motivates the involvement of prediction skills typical of action that, again, seems to be interwound with perception.

Having described all the regularization terms necessary to the well-posedness of the learning problem, we can introduce the following functional.

$$S\left(\phi ,v\right)=A\left(\phi ,v\right)+\lambda_{E}E\left(\phi ,v\right)+\lambda_{R}R\left(\phi ,v\right)$$ (22)

where *A*(***ϕ***, ***v***) is the direct generalization to feature groups of Eq. 10, that is $A\left(\phi ,v\right)=\frac{1}{2}\sum_{i=1}^{n}\int_{\Omega \times \left[0,T\right]}|\partial_{t}\phi^{i}+v\cdot \nabla \phi^{i}{|}^{2}d\mu \left(x,t\right)$ . Here, *λ*_*R*_ and *λ*_*E*_ > 0 are the regularization parameters. Learning to see means to discover the indissoluble pair (***ϕ*** ^⋆^, ***v*** ^⋆^) such that

$$\left(\phi^{⋆},\nu^{⋆}\right)=\underset{\left(\phi ,\nu \right)}{\arg\;\min}S\left(\phi ,\nu \right).$$ (23)

Basically, the minimization is expected to return the pair (***ϕ***, ***v***), whose terms should nominally be conjugated. The case in which we reduce to consider only the brightness, that is when the only ***ϕ*** is *I*, corresponds with the classic problem of optical flow estimation. Of course, in this case the term *R* is absent and the problem has a classic solution. Another special case is when there is no motion, so as the integrand in the definition Eq. 10 of *A* is simply null *∀i* = 1, *…*, *n*. In this case, the learning problem reduces to the unsupervised extraction of features ***ϕ***.

The learning of *ψ*_*i*_ can be based on a formulation that closely resembles what has been done for *φ*_*i*_, for which we have already considered the regularization issues. In the case of *ψ*_*i*_ we can get rid of the trivial constant solution by minimizing.

$$I_{\psi }=\overset{n}{\underset{i=1}{\sum }}\int_{\Gamma }\left(1-\psi_{i}\left(x,t\right)\right)\varphi_{i}\left(x,t\right)\;d\mu \left(x,t\right),$$ (24)

which comes from the p-norm translation (Gori and Melacci, 2013; Gnecco et al., 2015) of the logic implication *φ*_*i*_ ⇒ *ψ*_*i*_. Here we are assuming that *φ*_*i*_, *ψ*_*i*_ range in [0, 1], so as whenever *φ*_*i*_ gets close to 1, it forces the same for *ψ*_*i*_. This yields a well-posed formulation thus avoiding the trivial solution.

As far as the *χ* are concerned, like for *ψ*, we ask for the minimization of

$$I_{\chi }=\overset{n}{\underset{k=1}{\sum }}\int_{\Gamma }\left(1-\chi_{\kappa }\left(x,t\right)\right)\psi_{\kappa }\left(x,t\right)\;d\mu \left(x,t\right),$$ (25)

that comes from the p-norm translation of the logic implication *ψ*_*κ*_ ⇒ *χ*_*κ*_. While this regularization term settles the value of *χ*_*κ*_ on the corresponding *ψ*_*κ*_, notice that the motion invariance condition (18) does not assume any privilege with respect to the *firing feature ψ*_*κ*_.

### 3.4 Deep Networks-Based Realization of Vision Fields

In the previous sections we discussed invariance properties of visual features that lead to model the processes of computational vision as transport equations on the visual fields, see Eqs 9, 15, 17, 18. Some of those properties are based on the concept of consistency under motion, others lead to a generalization of the concept of affordance. In this section we will discuss how the features *φ*, *ψ* and *χ*, along with the velocity fields *v*_*φ*_, can be represented in terms of neural networks that operate on a visual stream and how the above theory can be interpreted in a classical framework of machine learning.

The first step we need to perform consists in moving to a discretized frame spatial support $\Omega^{⋄}=\left\{\left(i,j\right)\in N:0<i\leq w,\;0<i\leq h\right\}$ . As a consequence, the fields *φ*_*i*_ and the velocities can be viewed as vector-valued functions of time $t\mapsto \varphi_{i}\left(t\right)\in R^{\Omega^{⋄}}$ and $t\mapsto v_{\varphi_{i}}\left(t\right)\in {\left(R^{\Omega^{⋄}}\right)}^{2}$ ; similarly the discretized brightness can be seen as a map $t\mapsto I\left(t\right)\in R^{\Omega^{⋄}}$ .^3^ Then, features *φ*_*i*_ (and similarly the fields *ψ* and *χ*) can be modelled as neural networks $\Phi_{i}:R^{N}\times R^{\Omega^{⋄}}\to R^{\Omega^{⋄}}$ that given the brightness *I* at a certain instant and a set of *N* weights $w_{\Phi_{i}}\in R^{N}$ yield the value $\Phi_{i}\left(I,w_{\Phi_{i}}\right)$ of *φ*_*i*_. Similarly, the velocities *v*_*φ*_ can be estimated by a neural network $V_{i}:R^{M}\times R^{\Omega^{⋄}}\times {\left(R^{\Omega^{⋄}}\right)}^{2}\to {\left(R^{\Omega^{⋄}}\right)}^{2}$ that takes as inputs the temporal partial derivative $\dot{I}$ (that is the discrete version of the term *∂*_*t*_ *I*), the discrete spatial gradient ∇*I* of *I* and a given a set of *M* weights $w_{v_{i}}$ in order to predict $V_{i}\left(w_{v_{i}},\dot{I},\nabla I\right)$ as the velocity field $v_{\varphi_{i}}$ .

It should be noted that, within this framework, the learning problem for the fields *φ*, *χ*, *η* and *v*_*φ*_, that is based on the principles described in this paper and that is defined by the optimization problem of the form described in Eq. 23, becomes a finite-dimensional learning problem on the weights of the neural models.

**FIGURE 6:**
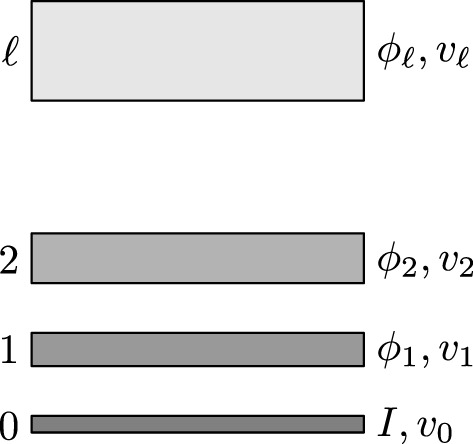
Different visual features along with the corresponding velocity fields.

Thus, learning will be affected by the structure (i.e. the architecture) of the network that we choose. Recent successes of deep learning within the realm of computer vision suggest that natural choices for Φ would be Deep Convolutional Neural Networks (DCNN). More precisely, the features extracted at level *ℓ* of a DCNN can be identified with a group of Φ_*i*_; in this way we are establishing a hierarchy between features that, in turn, suggests a natural way in which we could perform the grouping operation that we discussed in Section 3.1. In this way, features that are at the same level of a CNN share the same velocity (see Figure 6). In the case of velocities, CNN-based architectures like the one employed by FlowNet (see Fischer et al. (2015)) have been already proven to be suitable to model velocity fields.

It is also important to bear in mind that the choice of a specific neural architecture has strong repercussions on the way the invariance conditions are satisfied. For instance, let us consider the case of Convolutional Networks together with the fundamental condition expressed by Eq. 9. In this case, since CNN are equivariant under translations, any feature that tracks a uniformly translating motion of the brightness will automatically satisfy Eq. 9 with the same velocity of the translation of the input.

## 4 Discussion

In this paper we have proposed motion invariance principles that lead to discover identification features and more abstract features that are somewhat inspired to the notion of affordance. Those principles are expressed by motion invariance equations that characterize the interesting visual field interactions. The conjunction with features *φ* leads to believe that those features and their own velocities represent an indissoluble pair. Basically, the presence of a visual feature in the frame spatial support corresponds with its own optical flow, so as they must be jointly detectable. Apparently, in case of a visual agent without relative movement with respect to an object, this sounds odd. What if the object is fixed? Interestingly, foveate animals always experiment movement in the frame of reference of their eyes, and something similar can be experimented in computers by the simulation of focus of attention Zanca et al. (2019), Faggi et al. (2020). Hence, apart from the case of saccadic movements, foveate animals, like haplorhine primates, are always in front of motion, and conjugation of features with the corresponding optical flow does not result in trivial conditions.

The overall field interaction of features and velocities leads to compose a more abstract picture, since in the extreme case of features that represent objects, as already pointed out, we see the emergence of the classic notion of affordance. Interestingly, the described mechanisms of field interaction go well beyond the connection with such a high-level cognitive notion. We can promptly realize that it is impossible to understand whether the discussed field interactions come from different objects or if they are in fact generated within the same object. Overall, the discussed field interactions represent a natural mechanism for transmitting information from the video by local mechanisms.

It has been shown that in order to get a well-posedness of the motion invariance problems of Eqs 9, 15, 17, 18 we need to involve appropriate regularization. In particular, the development of visual features *φ* requires the correspondent minimization of Eq. 21, that somewhat indicates the need of involving action together with perception. Indeed, visual perception coupled with gaze shifts should be considered the *Drosophila* of perception-action loops. Among the variety of active behaviors the organism can fluently engage to purposively act upon and perceive the world (e.g, moving the body, turning the head, manipulating objects), oculomotor behavior is the minimal, least energy, unit. The history of these ideas has been recently reviewed in Bajcsy et al. (2018). At that time, such computational approaches were pervaded by the early work of Gibson (1950) who proposed that perception is due to the combination of the environment in which an agent exists and how that agent interacts with it. He was primarily interested in optic flow that is generated on the frame spatial support when moving through the environment (as when flying) realizing that it was the path of motion itself that enabled the perception of specific elements, while disenabling others. That path of motion was under the control of the agent and thus the agent chooses how it perceives its world and what is perceived within it Bajcsy et al. (2018). The basic idea of Gibson’s view was that of the exploratory behaviour of the agent. It is worth noting that despite of the pioneering work of Aloimonos et al. (1988), Ballard (1991), and Bajcsy and Campos (1992), gaze dynamics has been by and large overlooked in computer vision. The current state of affairs is that most effort is spent on salience modelling Borji (2021), Borji and Itti (2013) as a tool for predicting where/what to look at (the tacit though questionable assumption is that, once suitably computed, salience would be predictive of gaze). Interestingly enough, and rooted in the animate vision approach, Ballard set out the idea of predictive coding Rao and Ballard (1999):

We describe a model of visual processing in which feedback connections from a higher-to a lower-order visual cortical area carry predictions of lower-level neural activities, whereas the feedforward connections carry the residual errors between the predictions and the actual lower level activities. When exposed to natural images, a hierarchical network of model neurons implementing such a model developed simple cell-like receptive fields. A subset of neurons responsible for carrying the residual errors showed endstopping and other extra-classical receptive field effects. These results suggest that rather than being exclusively feedforward phenomena, nonclassical surround effects in the visual cortex may also result from cortico-cortical feedback as a consequence of the visual system using an efficient hierarchical strategy for encoding natural images.

This idea has gained currency in recent research covering many fields from theoretical cognitive neuroscience (e.g., Knill and Pouget, 2004; Ma et al., 2006) to philosophy Clark (2013). Currently, the most influential approach in this perspective has been proposed by Friston (e.g., Feldman and Friston, 2010; Friston, 2010) who considered a variational approximation to Bayesian inference and prediction (free energy minimization, minimization of action functionals, etc).

The principles on visual feature flow introduced in this paper might also have an impact in computer vision, since one can reasonably believe that the proposed invariances might overcome one of the major current limitation of supervised learning paradigms, namely the need of a huge amount of labeled examples. This being said, deep neural networks, along with their powerful approximation capabilities, could provide us with the ideal computational structure to complete the theoretical framework here proposed.

## Data Availability

The original contributions presented in the study are included in the article/supplementary material, further inquiries can be directed to the corresponding author.

## Footnotes

1 Under the assumption $\text{rank}\nabla {\left(R,G,B\right)}^{\prime }=\text{rank}\left(\nabla {\left(R,G,B\right)}^{\prime }\left|-\partial {\left(R,G,B\right)}^{\prime }/\partial t\right)\right.$ .

2 For example, we can understand that a person is sitting on or standing up from a chair just considering a still image.

3 Here we are overloading the symbols *φ*, *v*_*φ*_ and *I* in order to avoid a cumbersome notation. In the previous sections *φ*, *v*_*φ*_ and *I* are functions defined over the spatio-temporal cylinder Ω × [0, *T*], here they are instead regarded as vector-valued functions of time only.
